# Study of the progeny of sorghum mutants obtained using the CRISPR/Cas9 genetic construct directed at inducing mutations in the α-kafirin k1C5 gene

**DOI:** 10.18699/vjgb-25-122

**Published:** 2025-12

**Authors:** L.A. Elkonin, G.A. Gerashchenkov, N.V. Borisenko, S.Kh. Sarsenova, V.M. Panin

**Affiliations:** Federal Centre of Agriculture Research of the South-East Region, Saratov, Russia; Institute of Biochemistry and Genetics – Subdivision of the Ufa Federal Research Centre of the Russian Academy of Sciences, Ufa, Russia; Federal Centre of Agriculture Research of the South-East Region, Saratov, Russia; Federal Centre of Agriculture Research of the South-East Region, Saratov, Russia; Federal Centre of Agriculture Research of the South-East Region, Saratov, Russia

**Keywords:** Sorghum bicolor, CRISPR/Cas, alfa-kafirins, in vitro protein digestibility, endosperm, Sorghum bicolor, CRISPR/Cas, альфа-кафирин, перевариваемость in vitro, эндосперм

## Abstract

Site-directed mutagenesis using genetic constructs carrying the CRISPR/Cas system is an effective technology that is actively used to solve a variety of problems in plant genetics and breeding. One of these problems is to improve the nutritional value of grain sorghum, a high-yielding heat- and drought-tolerant cereal crop that is becoming increasingly important in the conditions of climate aridization. The main reason for the relatively low nutritional value of sorghum grain is the resistance of its storage proteins, kafirins, to proteolytic digestion. We have previously obtained mutants with improved kafirin in vitro digestibility using the CRISPR/Cas technology in grain sorghum variety Avance. The nucleotide sequence of one of the genes (k1C5) of the gene family encoding the signal polypeptide of 22 kDa α-kafirin was used as a target. The aim of this study was to investigate the manifestation of the main agronomically-important traits in the progeny of these mutants and inheritance of high in vitro protein digestibility, and also sequencing nucleotide sequences encoding the 22 kDa α-kafirin signal polypeptide in a number of plants from the T0 generation and their T1 progeny. It was revealed that four of the six studied T0 plants, as well as their progeny, had the same mutation: a T→C substitution in the 23rd position of the nucleotide sequence of the k1C5 gene encoding the signal polypeptide, which led to a substitution of the coding triplet CTC→CCC (Leu→Pro). This mutation is located off-target, 3’ from the PAM sequence. It is suggested that this mutation may have arisen as a result of Cas9 nuclease errors caused by the presence of multiple PAM sequences located close to each other. It was found that the progeny of two of the three studied mutants (T2 and T3 families), grown in the experimental field conditions, differed from the original variety by a reduced plant height (by 12.4–15.5 %). The peduncle length, 1,000-grain mass, and grain mass per panicle did not differ from the original variety, with the exception of the progeny of the 2C-1.2.5b mutant, which had a reduced grain yield per panicle. Unlike the original variety, plants from the T2 and T3 generations had kernels with a modified type of endosperm (completely floury, or floury with inclusions of vitreous endosperm, or with a thin vitreous layer). The level of grain protein digestibility in the progeny of mutants 2C-2.1.1 #13 and 2C-1.2.5a #14 varied from 77 to 84 %, significantly exceeding the original variety (63.4 ± 2.3 %, p < 0.05). The level of protein digestibility from kernels with modified endosperm was higher than that of kernels with normal vitreous endosperm (84–93 %, p <0.05). The reasons for the variation in endosperm texture in the progeny of the mutants and its relationship with the high digestibility of kafirins are discussed.

## Abstract (ru)

Сайт-направленный мутагенез посредством генетических конструкций, несущих систему CRISPR/Cas, считается эффективной технологией, активно применяемой для решения самых разных задач генетики и селекции у многих возделываемых культур. Зерновое сорго – уникальная по своей засухоустойчивости и жаростойкости злаковая культура, служащая источником кормов и пищевого зерна в аридных регионах земного шара, приобретающая все большее значение в условиях глобального потепления климата. Одной из основных задач селекции сорго явля- ется улучшение сравнительно низкой питательной ценности зерна, обусловленной в том числе устойчивостью его запасных белков – кафиринов – к протеолитическому расщеплению. Нами ранее путем использования технологии CRISPR/Cas у зернового сорго сорта Аванс были получены мутанты с улучшенной перевариваемостью кафиринов в системе in vitro. При этом в качестве мишени был выбран один из членов генного семейства, кодирующего 22 кДа α-кафирин, а именно ген k1C5. Цель данного исследования – изучение потомства полученных нами ранее растений, несущих мутации в гене k1C5, отличающихся улучшенной перевариваемостью белков зерна в системе in vitro, а имен- но: анализ наследования высокой перевариваемости, проявления основных селекционно ценных признаков, а также выявление структуры нуклеотидной последовательности, кодирующей сигнальный полипептид 22 кДа α-кафирина у ряда растений из поколения Т0 и их потомства T1. Обнаружено, что у четырех из шести изученных растений Т0, а также у их потомства присутствует одна и та же мутация: замена Т→С в 23-й позиции нуклеотидной последователь- ности гена k1C5, кодирующей сигнальный полипептид, которая привела к замене кодирующего триплета CTC→CCC (Leu→Pro). Эта мутация располагается за пределами выбранной мишени, в направлении 3’ от последовательности PAM. Высказывается предположение, что данная мутация могла возникнуть в результате ошибок нуклеазы Cas9, обусловленных наличием нескольких последовательностей PAM, расположенных близко друг к другу. Установлено, что потомство двух из трех изученных мутантов (семьи T2 и Т3), выращенное в условиях опытного поля, отличалось от исходного сорта сниженной высотой растения (на 12.4–15.5 %). Длина цветоножки, масса 1000 зерен и масса зерна с метелки не отличались от исходного сорта, за исключением потомства мутанта 2С-1.2.5b, у которого урожай зерна с метелки был снижен. В отличие от исходного сорта, у растений из поколений Т2 и Т3 присутствовали зерновки с модифицированным типом эндосперма (полностью мучнистым, либо с вкраплениями стекловидного эндосперма, либо с тонким стекловидным слоем). Уровень перевариваемости белков зерна в потомстве мутантов 2С-2.1.1 № 13 и 2С-1.2.5a № 14 варьировал от 77 до 84 %, значимо превышая показатель исходного сорта (63.4 ± 2.3 %, p < 0.05). Уровень перевариваемости белков из зерновок с модифицированным эндоспермом был выше, чем у зерновок с обычным стекловидным эндоспермом (84–93 %, p < 0.05). Обсуждаются причины варьирования текстуры эндоспер- ма у потомства полученных мутантов и ее связь с высокой перевариваемостью кафиринов

## Introduction

Modifying the nucleotide sequences of genes using the CRISPR/Cas genome editing technology is one of the most powerful tools in plant genetics and breeding (Zhu et al., 2020; Gao, 2021; Saini et al., 2023). In recent years, the CRISPR/ Cas technology has been intensively used in many cultivated plant species, including sorghum, a unique drought- and heatresistant cereal crop that serves as a source of feed and food grain in arid regions of the globe. Despite the fact that sorghum is one of the most difficult cereal species to transform, many studies have appeared on sorghum genome editing using the CRISPR/Cas technology, which have been summarized in a number of reviews (Balakrishna et al., 2020; Parikh et al., 2021; Wong A.C.S. et al., 2022).

One of the most actual problems in sorghum breeding is improving the digestibility of grain storage proteins. Sorghum grain contains a significant amount of protein (on average 10–12 %, and in some lines up to 16–18 %), represented by different classes of kafirins (α, β, γ, δ), related to alcoholsoluble proteins – prolamins, which make up to 70–80 % of the total protein content, and non-kafirin proteins, the composition of which is poorly studied (Bean et al., 2018). Different classes of kafirins differ in their molecular weight and amino acid composition, and are encoded by different genes. An important feature of kafirins is their resistance to proteolytic digestion. As a result, the level of in vitro grain protein digestibility in the vast majority of varieties and hybrids does not exceed 40–60 % (Wong J.H. et al., 2010; Elkonin et al., 2013; Duressa et al., 2018). Such resistance of kafirins to proteolytic digestion also reduces the digestibility of starch, since undigested kafirins prevent complete amylolytic cleavage of starch granules (Zhang, Hamaker, 1998; Ezeogu et al., 2005; Wong J.H. et al., 2009).

The resistance of kafirins to protease digestion is multifactorial (see reviews: Belton et al., 2006; Duressa et al., 2018). These factors include the chemical structure of kafirins, which are rich in sulfur-containing amino acids (especially γ- and β-kafirins) capable of forming intra- and intermolecular crosslinks, which are believed to prevent the proteolytic cleavage of kafirins; the interaction of kafirins with polyphenols, which inhibit protease activity. An important factor is the spatial organization of different kafirins in the protein bodies of endosperm cells. In the early stages of endosperm development, γ- and β-kafirins are synthesized and deposited in protein bodies developing in the endoplasmic reticulum. Alphakafirin, synthesized at later stages of endospermogenesis, is deposited inside protein bodies, pushing γ-kafirin to the periphery, which forms a kind of “shell” that is difficult for proteases to digest (De Mesa-Stonestreet et al., 2010; Duressa et al., 2018).

As a result of the study of mutants with impaired synthesis of kafirins obtained using RNA interference (see review: Elkonin et al., 2021), it was found that partial suppression of kafirin synthesis significantly improves the digestibility of grain proteins and promotes the synthesis of other proteins with higher nutritional value. In this regard, targeted induction of mutations in the genes encoding kafirin synthesis can contribute to the production of new sorghum lines with improved digestibility of grain proteins, which, unlike lines carrying the genetic construct for RNA silencing, will be devoid of transgenes

In recent years, several studies have been published reporting successful editing of α-, β-, and γ-kafirin genes (Li A. et al., 2018; Massel et al., 2022, 2023; Elkonin et al., 2023; Li X. et al., 2024). Most of these studies targeted nucleotide sequences encoding signal polypeptides responsible for the deposition of α- and γ-kafirins in the protein bodies of endosperm cells (Li A. et al., 2018; Elkonin et al., 2023; Li X. et al., 2024). These mutants had improved digestibility of grain proteins, in contrast to mutants with mutations in the β-kafirin gene structure (Massel et al., 2023).

The aim of this study was to explore the progeny of previously obtained plants carrying mutations in the k1C5 gene, characterized by improved digestibility of grain proteins; namely, to study the inheritance of high digestibility, the manifestation of the main agronomically important traits, and to identify the structure of the nucleotide sequence of the k1C5 gene encoding the 22 kDa α-kafirin signal polypeptide

## Materials and methods

Material and growing conditions. The progenies of T1 plants with high in vitro protein digestibility, which were obtained from the T0 mutants 2C-2.1.1 [T1 #11 (86.6 % digestibility) and T1 #13 (86.7 %)], T0 2C-1.2.5a [T1 #11 (92.4 %) and T1 #14 (77.3 %)], and T0 2C-1.2.5b [T1 #14 (91.8 %)], were studied. These mutants were obtained in genome editing experiments with grain sorghum cv. Avance using the binary vector p2C containing the Cas9 endonuclease gene and gRNA targeted at the nucleotide sequence of the k1C5 gene encoding the 22 kDa signal polypeptide of α-kafirin (Elkonin et al., 2023). The selected T1 plants with high protein digestibility did not contain the CRISPR/Cas genetic construct (Elkonin et al., 2023). The studied progenies (T2 and T3 generations) were grown in the experimental field of the Federal Centre of Agriculture Research of the South-East Region (Saratov, Russia). Plants were grown in 4-m rows with 70 cm row spacing, with a plant density of 6 plants per 1 m. The panicles of all plants were carefully bagged in parchment bags before flowering. The following traits were analyzed: plant height, peduncle length, 1,000-grain mass, grain yield per panicle, endosperm type, and in vitro digestibility of grain proteins. In each family, 10–20 plants were studied.

Grain protein digestibility. To study the digestibility of grain proteins, the method of treating whole-milled flour with pepsin was used (Aboubacar et al., 2001; Wong J.H. et al., 2009). In this case, a weighed sample of flour (60 mg) was incubated in 1 ml of 0.15 % pepsin solution (Sigma-Aldrich, P7000; 250 units/mg) in 0.1 M potassium phosphate buffer (pH 2.0) at 37 °C on a shaker (70 rpm).

A method based on scanning the electrophoretic spectra of proteins obtained in SDS-PAGE was used for quantitative assessment of digestibility (Aboubacar et al., 2001; Nunes et al., 2004; Wong J.H. et al., 2009; Elkonin et al., 2013). For this purpose, flour samples after pepsin digestion, as well as control samples incubated in potassium phosphate buffer without the addition of pepsin, were centrifuged at 13,000 rpm; the pellet was incubated in extraction buffer (0.0125 M Na2B4O7, pH 10.0) under reducing conditions (1 % SDS, 2 % 2-mercaptoethanol) at room temperature for 2 h, after which it was boiled (100 °C) for 5 min. Samples were centrifuged at 13,000 rpm and separated by SDS-PAGE on 12.5 % (w/v) polyacrylamide gel according to a modified Laemmli method (Laemmli, 1970). 15 μl of extract were added to each lane. Separation was monitored using protein molecular weight markers, 10–200 kDa (Servicebio, G2058, Wuhan, Hubei, China). Gels were stained with Coomassie R-250. After electrophoresis, gels were scanned using the ChemiDoc system (Bio-Rad Laboratories, Hercules, CA, USA); the protein amount was estimated using Image Lab 6.1 software (Bio-Rad). Digestibility indices were calculated as the percentage difference between the protein volume in the control sample and the digested sample, relative to the control sample. The previously obtained Avance-1/18 mutant with a genetic construct for RNA silencing of the gKAF1 gene was used as a standard of high in vitro digestibility (Elkonin et al., 2021). Experiments were performed in duplicate.

Endosperm texture. The endosperm texture was determined on cross-sections of mature kernels, which were made using a sharp scalpel. The following types of endosperm were distinguished: normal with a thick vitreous layer and modified, which included floury, floury with blurred vitreous endosperm, and floury with a thin rim of vitreous endosperm. Forty kernels were analyzed from each plant

Sequencing of the k1C5 gene nucleotide sequence. To identify mutations, PCR amplicons of the k1C5 gene (primers F: 5′-TTGCCAGGGCTAGTTGACTG-3′ and R: 5′-AGGCTTTGATCCACATGAGCA-3′) were cloned into the pAL2-T vector (Eurogen, Russia). Sanger sequencing was performed by Syntol (Moscow, Russia) on an ABI 3130 genetic analyzer (sequencing primer: 5′-TTGCCAGGGC TAGTTGACTG-3′). Mutations in the sequenced amplicons were identified using Chromas (https://www.technelysium. com.au) and SnapGene Viewer 5.2.4 (https://www.snapgene. com) computer programs.

Methods of biological statistics. To assess differences in in vitro protein digestibility of the studied samples, one-way ANOVA was performed using the AGROS software package, version 2.09 (S.P. Martynov, Institute of Genetics of the Russian Academy of Sciences), and Duncan’s Multiple Comparisons Test. Differences in the manifestation of morphometric traits between mutant families and the original variety were assessed using Student’s t-test.

## Results

**Sequencing of the nucleotide sequence encoding the 22 kDa signal polypeptide of α-kafirin of the k1C5 gene**

**Fig. 1.**
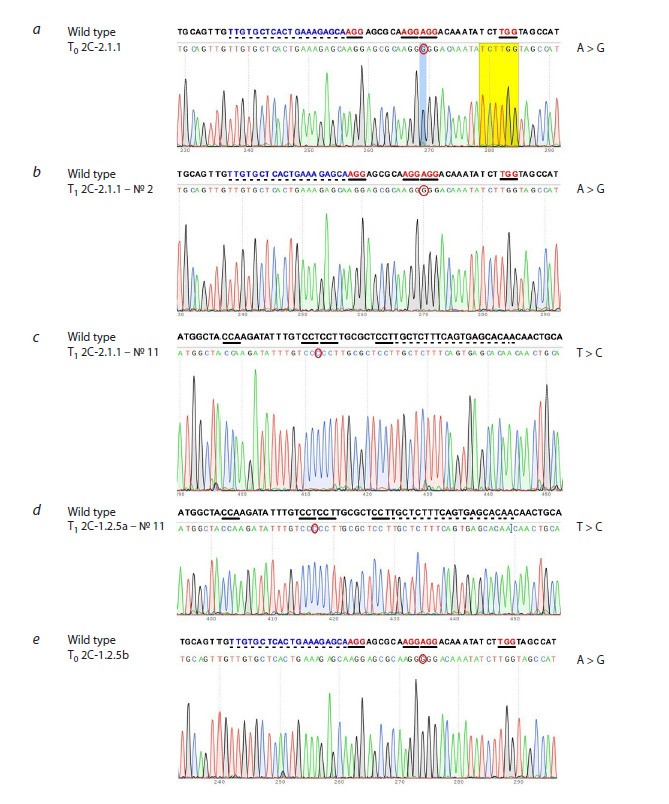
Results of sequencing of the nucleotide sequences encoding 22 kDa α-kafirin signal polypeptides in Т0 2C-2.1.1 (a) and its Т1 progeny 2C-2.1.1, plant #2 (b) and plant #11 (c); Т1 2C-1.2.5a, plant #11 (d); Т0 2C-1.2.5b (e). a, b, e – R-chain; c, d – F-chain. PAM sequences are underlined with a solid line; the target sequence is dashed. The nucleotide sequence encoding the 22 kDa α-kafirin signal polypeptide was taken from the Phytozome website, https://phytozome.jgi.doe.gov: Sobic.005G193100, Chr05: 67654898–67655764.

Sequencing of the nucleotide sequence encoding the 22 kDa α-kafirin signal polypeptide of one of the genes of the k1C gene family (k1C5) in two plants from the progeny of the T0 2C-2.1.1 mutant, #2 and #11 (T1 generation), characterized by improved digestibility of endosperm proteins (86 and 87 %, respectively), showed that they have the same mutation: a substitution of the 23rd nucleotide, counted from the 5′-end of the nucleotide sequence of the signal polypeptide (in the F-chain: T→C; in the R-chain: A→G) (Fig. 1b, c). Sequencing of a similar sequence in the original T0 mutant showed that the same mutation was also present in the parental T0 plant (Fig. 1a). In silico analysis showed that this mutation leads to a substitution of the coding triplet CTC→CCC, which should result in a substitution of the eighth amino acid of the α-kafirin signal polypeptide, namely, in the substitution of leucine, an aliphatic non-polar hydrophobic amino acid, for proline, a heterocyclic less hydrophobic amino acid that causes a bend in the α-helix of the protein. Such a substitution could change the structural and functional properties of the polypeptide and, as a consequence, the nature of α-kafirin deposition in protein bodies, and thereby affect their digestibility

Sequencing of a similar amplicon in one of the T1 plants from the progeny of another T0 mutant 2C-1.2.5a #11, characterized by improved protein digestibility (92 %), also revealed the presence of a T→C mutation at the same site of the nucleotide sequence encoding the signal polypeptide (Fig. 1d). Remarkably, we identified the same mutation by sequencing the DNA of another T0 plant 2C-1.2.5b (Fig. 1e), regenerated from the same callus as 2C-1.2.5a. Previously, we found the same mutation in the T0 plant 2C-1.2.9, while this mutation was absent in two other T0 plants (1C-2.1.1 and 2C-1.2.4) (Elkonin et al., 2023).

Thus, four out of the six T0 plants studied have the same mutation: a T→C substitution at position 23 of the nucleotide sequence of the k1C5 gene, and this mutation is inherited in the T1 generation.

**Manifestation of agronomically important traits**

**Fig. 2.**
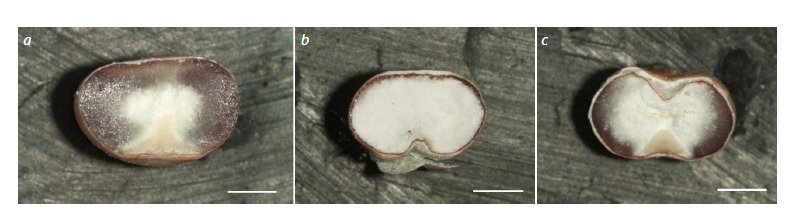
Cross-sections of the kernels of the mutant 2C-1.2.5a (plants from family 208/13). a – normal vitreous endosperm, b – floury endosperm, c – endosperm with a blurred vitreous layer. Scale bar 1 mm.

An analysis of the manifestation of the main agronomically important traits in the progeny of mutants with improved digestibility of endosperm proteins obtained by us earlier (Elkonin et al., 2023) revealed that in the T2 generation, two of them, 2C-1.2.5a and 2C-1.2.5b (families 203/23 and 200/23), had reduced plant height compared to the original cv. Avance, by 12.4–15.5 %, respectively (Table 1). The reduced plant height in the 2C-1.2.5a mutant was also inherited in the T3 generation (by 5.5 %, family 208/23). The length of the peduncle (protrusion of the paniculate internode) did not differ in the progeny of the mutants and the original cv. Avance. The 1,000-grain mass and grain yield per panicle in all families also did not differ from the original cv. Avance, with the exception of the progeny of the 2C-2.1.1 mutant (T2 195/23 family), which had larger and heavier grains, and the progeny of the 2C-1.2.5b mutant (T2 200/23 family), which had reduced grain yield per panicle. In plants of all the studied families, most kernels had endosperm of the normal vitreous type, characteristic of the original cv. Avance. However, almost all fami- lies contained plants that had kernels with a floury endo- sperm, or with a blurred or thin vitreous layer (Fig. 2), i. e., endosperm types characteristic of mutants with impaired kafirin synthesis (Elkonin et al., 2021). The proportion of such kernels in some plants from families 197/23 (T3 generation of the mutant 2C-2.1.1) and 208/23 (T3 generation of the mutant 2C-1.2.5a) reached 35–40 % (Table 1). Often, such kernels were smaller in size compared to kernels with normal vitreous endosperm.

**Table 1.**
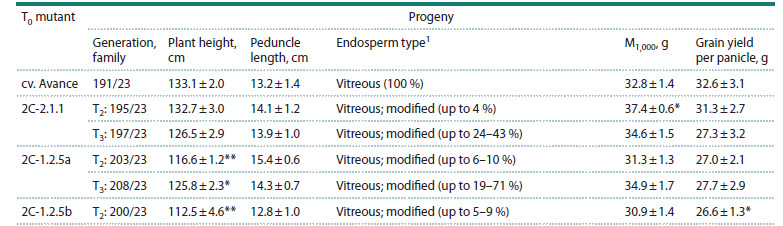
Manifestation of agronomically valuable traits in the progeny of sorghum mutants obtained using the CRISPR/Cas genetic construct targeting the 22 kDa α-kafirin gene (k1C5) *, ** Differs from the original cv. Avance at p <0.05 and p < 0.01, respectively. 1 The proportion of kernels with a different endosperm type in different plants from the family.

**Fig. 3.**
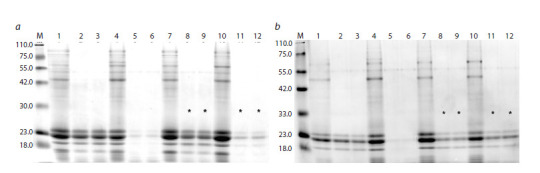
Electrophoretic spectra of proteins from flour of sorghum kernels from different generations of the mutant 2C-2.1.1 obtained by site-directed mutagenesis of the k1C5 gene. a: plant #197-9/23 from the T3 generation (lanes 7–12) (experiment 03.09-2, see Table S1)1. b: plant #195-3/23 from the T2 generation (lanes 7–12) (experiment 13.08-1, see Table S1). On both plates: 1–3 – original cv. Avance; 4–6 – mutant with RNA silencing of the gKAF1 gene (Elkonin et al., 2021) (standard of high level of in vitro protein digestibility); 7–9 – kernels with normal vitreous endosperm; 10–12 – kernels with floury endosperm; 1, 4, 7, 10 – control samples (without pepsin treatment); 2, 3, 5, 6, 8, 9, 11, 12 – samples after pepsin treatment (two replicates for each sample); M – molecular weight markers (Servicebio, G2058). The spectra of samples characterized by significantly higher digestibility compared to the Avance variety (Table 2, Table S1) are marked with asterisks.


Analysis of grain protein digestibility in the progeny of mutants 2C-2.1.1 #13 and 2C-1.2.5a #14 (both from T1) showed that increased values of this trait, compared to the original cv. Avance, were manifested in plants from generations T2 and T3 (Fig. 3). For example, in the progeny of mutant 2C-2.1.1 #13 (Table 2, families T2 195/23 and T3 197/23), as well as mutant 2C-1.2.5a #14 (family T3 208/23), the digestibility level reached 77–84 %, exceeding the original cultivar by 10–20 % (p <0.05), while a significantly higher digestibility level was observed in kernels with a normal vitreous type of endosperm, characteristic of the original cultivar. At the same time, the level of protein digestibility from kernels with floury or blurred vitreous endosperm was significantly higher than that of kernels with normal vitreous endosperm, reaching 84–93 % and significantly exceeding the level of digestibility in the original cultivar (p < 0.05), which did not have such kernels.

Supplementary Materials are available in the online version of the paper: https://vavilov.elpub.ru/jour/manager/files/Suppl_Elkonin_Engl_29_8.pdf


**Table 2.**
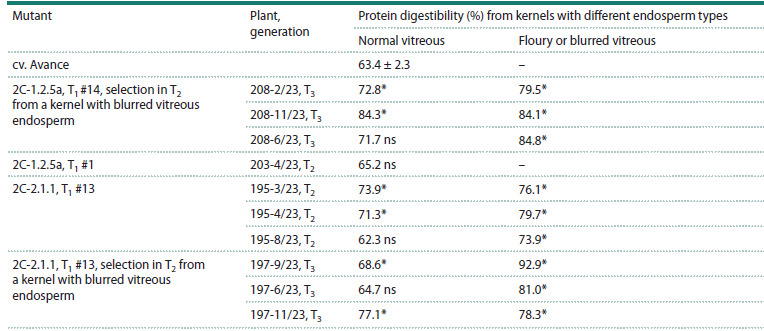
In vitro digestibility of flour proteins from kernels with different endosperm types in some plants from the progeny of sorghum mutants obtained by site-directed mutagenesis of the k1C5 gene Note. * Differs from the original cv. Avance at p < 0.05, in accordance with the F-criterion (Table S1); ns – no significant differences from cv. Avance when analyzing the corresponding SDS-PAGE.

## Discussion

The CRISPR/Cas technology is considered one of the most effective tools for inducing mutations at strictly defined loci of plant genome. However, in some cases, the precision of editing gene nucleotide sequences using the CRISPR/Cas9 system may be flawed (Sturme et al., 2022; Guo et al., 2023; Movahedi et al., 2023).

In our experiments, four out of six studied T0 plants had the same mutation: a T→C substitution at position 23 of the k1C5 gene nucleotide sequence. This mutation is located outside the selected target, 3′-end to the PAM (protospacer-adjacent motif) sequence, and is therefore off-target. A detailed analysis of the nucleotide sequence of this region of the k1C5 gene revealed the presence of several PAM sites located close to each other: two 5′-AGG and one 5′-TGG (Fig. 1). It is possible that due to such proximity, the Cas9 nuclease could make errors and introduce breaks between the two PAM sites: AGG ↓ AGG. Therefore, one of the reasons for the occurrence of off-target mutations, as our data show, may be a significant number of closely located PAM sites. Similar examples of off-target Cas9 activity, where a mutation occurs in the target gene but outside the chosen target, were previously found in a study editing the Phytoene desaturase (PDS) gene in two cassava varieties (Manihot esculenta Crantz) (Odipio et al., 2017). Notably, in another work in sorghum on editing the nucleotide sequence encoding the signal polypeptide of the γ-kafirin gene using a CRISPR/Cas9 genetic construct, mutations occurred not at the canonical site, between the 3rd and 4th nucleotides 5′-end to the PAM, but after the 15th nucleotide of the target and beyond, 5′-end to the PAM site, but within the target gene (Li X. et al., 2024). These facts raise questions about the accuracy of genome editing using Cas9 nuclease in sorghum.

Another important point worthy of discussion is the decrease in the level of kafirin digestibility in the progeny of the mutants we obtained. Previously, we found a significant increase in the level of grain protein digestibility in a number of mutants obtained in the T1 generation: up to 80–87 % in the 2C-2.1.1 mutant, up to 86 and 92 % in the 2C-1.2.5b and 2C-1.2.5a mutants, respectively (Elkonin et al., 2023). In the T3 generation, the digestibility level decreased to 68–74 % in the 2C-2.1.1 mutant and 72–84 % in the 2C-1.2.5a mutant, significantly exceeding, however, the same indicator in the original cv. Avance (Table 2, Table S1); in the plants from the progeny of the 2C-1.2.5b mutant, there were no significant differences from cv. Avance

A possible reason for such a decrease in digestibility may be different growing conditions of the plants: the T1 generation was grown in a climate chamber under conditions of regular watering and high air humidity, while the T3 plants were grown in an experimental field plot. It is known that under drought stress conditions, the digestibility of sorghum grain proteins is significantly reduced in some cultivars (Impa et al., 2019). In addition, a possible compensatory increase in the expression of other genes controlling the synthesis of kafirins, which led to the restoration of their content and a decrease in the level of digestibility of grain proteins, cannot be ruled out. Such a compensatory increase in the content of γ-kafirin was previously found in sorghum mutants with impaired synthesis of β-kafirin, which restored the overall balance of kafirins in the grain and did not lead to an improvement in the digestibility index (Massel et al., 2023).

Of particular interest is the variation in endosperm texture in mutants from different generations. In T0 plants, the formation of kernels with impaired development of vitreous endosperm was observed (Gerashchenkov et al., 2021). Such kernels are characteristic of sorghum mutants with partially suppressed kafirin synthesis and high protein digestibility (see reviews: Duressa et al., 2018; Elkonin et al., 2021). In T1 plants, kernels with normally developed vitreous endosperm and high protein digestibility were formed, which was an unusual phenomenon, given the close correlation between high digestibility and floury endosperm in sorghum (Duressa et al., 2018). In the T2 and T3 generations, plants from a number of families again had kernels with a modified type of endosperm (floury or with a thin, often blurred vitreous layer along the periphery of the endosperm), which were distinguished by a significantly higher level of digestibility (Table 2). As a result of the selection of such kernels, we obtained two T3 families, 208/23 and 197/23, in which plants contained kernels with normal vitreous endosperm with a higher level of protein digestibility than in the original cultivar. Such variations in the endosperm texture may be a consequence of the influence of environmental conditions on the expression of the induced mutation, or another mutation that we have not yet identified affects the modification of the endosperm type. A more definitive conclusion can be made in the future as a result of additional studies.

## Conclusion

In summary, as a result of studying the progeny of sorghum mutants obtained using the CRISPR/Cas9 genetic construct aimed at inducing mutations in the k1C5 gene encoding the synthesis of α-kafirin, we identified two T3 families, 208/23 and 197/23, in which the plants contained kernels with normal vitreous endosperm and a higher level of grain protein digestibility in comparison with the original cultivar (up to 72–84 %, compared to 62–64 % in the original cv. Avance). Plants from these families do not have significant differences in the manifestation of agronomically valuable traits from the original cultivar, with the exception of reduced height (by 5–15 %), and do not contain the CRISPR/Cas genetic construct. Four of the six T0 plants studied harbor the same mutation: a T→C substitution at position 23 of the k1C5 gene sequence, and this mutation is inherited by the T1 generation. This mutation is located 3′-end to the PAM sequence, and may be a consequence of off-target Cas9 activity, in which the mutation occurs in the target gene but off-target due to the presence of multiple PAM sites located close to each other.

## Conflict of interest

The authors declare no conflict of interest.
